# The Smallpox Vaccine in Latin America: A New Approach (1801–1804)

**DOI:** 10.3390/medicina59061093

**Published:** 2023-06-05

**Authors:** Antonio Pérez Pérez, José Ramón Vallejo

**Affiliations:** 1 Área de Anestesiología y Reanimación, Hospital Universitario Puerta del Mar, E-11009 Cádiz, Spain; 2 Departamento de Anatomía Patológica, Biología Celular, Histología, Historia de la Ciencia, Medicina Legal y Forense y Toxicología, Facultad de Medicina, Universidad de Cádiz, E-11003 Cádiz, Spain

**Keywords:** smallpox, vaccine, epidemics, the Spanish Navy, overseas, Royal College of Medicine and Surgery of Cadiz, Medical–Surgical School of Cadiz, history of medicine

## Abstract

The Royal Philanthropic Vaccine Expedition is considered in the history of medicine as the first international health expedition aimed at the global elimination of a contagious disease: smallpox. However, the initiatives carried out in this way before the arrival of the Balmis Expedition, by surgeons from the Spanish Navy, are less well known. Thus, the main objective of this research work is to offer an overview of the different anti-variolic vaccination initiatives prior to the campaign financed by the Spanish crown from these health facilities. Using the heuristic and hermeneutic method, our article is based on primary sources contrasted with specialised literature. The results obtained are presented in a narrative style from each of the surgeons identified as decisive in the implementation of the vaccine, thus providing a divergent and unpublished historiographic approach. As the facts described show, before the arrival of Balmis the vaccine substance was introduced in those countries thanks to the initiative of various surgeons: in Puerto Rico by Francisco Oller; in Cartagena and Santa Marta in Colombia by Ángel Hidalgo; in Venezuela by Alonso Ruiz; in Cuba by Tomás Romay and Bernardo de Cózar; in the Viceroyalty of New Granada (Colombia) by Lorenzo Vergés; in Guatemala by Miguel José Monzón and José María Ledesma; in the Viceroyalty of New Spain by Alejandro García Arboleya and Antonio Serrano; in Peru by Pedro Belomo; in Río de la Plata by Cristóbal Martín de Montúfar; in the Chilean region of Coquimbo by José María Gómez; and in the Philippines by Cristóbal Regidor. Finally, it should be noted that these surgeons and the approach presented are part of a historiography based on the personal actions of professionals trained, for the most part, at the Medical–Surgical School of Cadiz.

## 1. Introduction

It was from the 18th century onwards that smallpox, in both Europe and America, saw an ostensible increase in virulence, coming to represent, for both civilian and medical authorities on both continents, a major public health concern, as it became, from the point of view of historical epidemiology, a truly “social disease”. Until then, as Haeser noted, “it was not the rarity of smallpox but rather its uninterrupted presence that made epidemiologists disinterested in it” [1]; however, since the previously mentioned century, the perception of smallpox changed radically among epidemiologists, as like the other miasma-related pathologies of the time, such as yellow fever or malaria, smallpox caused unexpected epidemics that were beyond any seasonal or any other type of forecast, regardless of age, gender or social rank, and decimated the affected populations, leaving significant physical and psychological scars among the survivors.

Precisely as a result of this social and medical concern, different preventive and control measures were developed against it, mainly in the form of different treatments, generally characterised by their lack of effectiveness: rules and regulations aimed at isolating those affected; the introduction of quarantines in seaports; and finally inoculation campaigns, which were later replaced by vaccination campaigns against the disease [2] (p. 154).

In Spain, unlike inoculation, vaccination against smallpox was quickly accepted. To understand this, we must take into account the fact that this disease also affected the Spanish Bourbon dynasty in a traumatic way; in this regard, it is worth remembering that Luis I, who had recently ascended to the Spanish throne, died a few days later from smallpox. In the same way, in 1788, the Infante Gabriel de Borbón, the tenth son of Carlos III, suffered from the same illness, as did his wife María Victoria Josefa de Braganza, and their newborn son, Carlos José Antonio; similarly, ten years later, the Infanta María Luisa Carlota, daughter of Carlos IV, suffered from this illness and although the doctors of the Chamber managed to cure her, they did not prevent her face from becoming disfigured, which caused alarm at the Madrid Court, to the extent that the monarch, on the proposal of Francisco Martínez de Sobral, agreed to inoculate the rest of the infants and other members of the Royal family; this preventive practice, however, was not without risk, leading, for example, to the serious convalescence of Prince Ferdinand and the well-known post-inoculation ophthalmia of María Amalia de Borbón, wife of the Infante Antonio Pascual. However, in spite of these consequences, the Spanish king valued this preventive technique positively, as it at least prevented death, which was the reason for the promulgation of the Royal Decree of 30 November 1798 [3], which generalised inoculation among his subjects.

In short, the monarchical acceptance of inoculation paved the way in Spain for the introduction, dissemination and generalisation of the vaccine discovered in 1796 by Edward Jenner.

In this way, the first Jenner vaccinations in Spain took place relatively soon after the publication in 1798 by the English doctor, specifically on 3 December 1800, by Francisco Piguillem in the Catalan town of Puigcerdà, using vaccinal fluid sent to him from Paris by François Colon [4] (p. 25).

Later, from Catalonia, the smallpox lymph was taken to Aranjuez and, almost immediately, to Madrid, although the initial results were not as expected, which is why it was necessary to wait until the arrival, on 22 April 1801, of a new consignment of smallpox fluid coming directly from the French capital, sent by the diplomat Luis de Onís, with which the medically trained Félix González, the Court doctor, achieved the expectations he had hoped for in the capital of the kingdom [5].

In these first trials for the introduction of Jenner’s vaccine in Spain, we cannot ignore the initiatives taken in its favour by doctors and surgeons from the Basque country and Navarre, such as, for example, Lope García de Mazarredo in Bilbao; Salvador María Bonor, José Antonio de Irízar and Vicente Lubet in San Sebastián; and Diego de Bances and Vicente Martínez in Navarre [6].

In other words, we can confirm that in general, unlike what happened with inoculation, Spanish doctors willingly accepted this new preventive remedy against smallpox for both scientific and ideological reasons, with Ignacio María Ruíz de Luzuriaga prominent in this order [7], as well as the surgeons of the Navy, Juan Manuel de Aréjula and José Sabater [4] (pp. 25–26).

In 1921, the Spanish philosopher Ortega y Gasset stated that “the groups that make up a State live together for something: they are a community of purposes, of desires, of great utilities. They do not live together to be together, but to do something together” [8] (p. 25). This is precisely the problem that we discuss in this research work when facing the unknown but no less certain reality that the introduction and propagation of the vaccine in overseas Spain had the privilege of being doubly established, thanks undoubtedly, on the one hand, to the contribution of resources from the metropolis itself but, on the other hand, to not indisputably neglecting those other means previously found in those same territories.

## 2. The Pandemic Overseas and the Need to Provide Another Historical Look

On 7 September 1806, the surgeon Francisco Xavier de Balmis was received at the Court of Madrid by King Carlos IV, as a true national hero [1] after having completed an undertaking considered in the annals of the history of medicine as an epic hitherto unparalleled worldwide, that is, the conclusion of the first global vaccination programme carried out to definitively eradicate smallpox, a contagious disease, characterised by its high morbidity and mortality rate, from all Spanish overseas territories.

However, despite this indisputable feat carried out in the nineteenth century by those Spanish expeditionaries, headed by Balmis, a person of the stature of Gregorio Marañón [9], the father of Spanish endocrinology, pointed out in 1948 that Balmis’s achievement did not lie in the feat of introducing and spreading the vaccine in America and the Philippines but, on the contrary, in becoming the archetypal representation of the enlightened and philanthropic Spanish spirit that illuminated European medicine of his time. However, what did Marañón really mean by this statement?

The constant epidemics of smallpox registered in the overseas Spanish provinces, particularly after 1800, urgently demanded the presence of the vaccine in those provinces. This lack of vaccine forced the Creole authorities and the doctors in favour of such a useful remedy to request it from the centres that had it before the arrival in 1804 of the Royal Philanthropic Expedition headed by Balmis in the New Continent, a reason that enables us to affirm a priori that the vaccine did not reach the overseas Spanish domains exclusively by the aforementioned Expedition.

We would like to point out that this very interesting subject has previously been treated by different groups of authors who have progressively distanced themselves from the historiographical tradition. Therefore, initially, the subject has been treated with varying success, but always partially by a small number of researchers, among whom we can mention, mainly, Clavijo y Clavijo in 1936 [10], Ramírez Martín in 1999 [1] and Balaguer Perigüell and Ballester Añón in 2003 [11]. Subsequently, a line of research has been generated, related to the participation of surgeons from New Spain in a general way or focusing especially on actions related to local surgeons or from an ideological point of view [12], as has also been conducted via analysis of the first stages of this disease in Spain [13].

For all these reasons, the main purpose of this work is to provide an overview of the different smallpox vaccination initiatives prior to the campaign funded by the Spanish crown under the direction of Francisco Xavier de Balmis, chamber surgeon to Carlos IV. In this way, we set out to clearly identify the protagonists and we wish to emphasise collaboration in the dissemination of the role played in the introduction of the smallpox vaccine by a large group of surgeons from the Royal Spanish Navy. It should be noted that in many cases these professionals were connected to the Royal College of Medicine and Surgery of Cadiz. In addition, we wish to raise the need for further local studies to better understand the events, and in particular the attitudes, values and implicit standards of the authorities and communities around the health policies of the time. For this reason, we are in line with more recent historical works on smallpox epidemics that raise the need for historians to build more complete contexts to better understand public health strategies, the limits of medical science and local responses [14,15,16,17].

Following this procedural philosophy, a section of results structured by subheadings has been made for each of its protagonists, that is, the surgeons of the Navy who have until now not been sufficiently recognised. Each of the identified surgeons allows a narrative construction of the events from another perspective. However, before moving on to the story itself we describe the method used to highlight the theoretical importance of classical historiography, as well as the archives and documents consulted so far, dispersed and immersed in a grey literature of difficult access.

## 3. Materials and Methods: The Epistemological and Historiographical Approach

This study corresponds, by its character, to a historical-medical research work; therefore, by its very nature, an intrinsically historiographic methodology has been used in its design. In this sense, and as a reminder, it should be noted that both the method and techniques of historical research have evolved over time according to new models and objects of study [18,19,20,21,22,23]. In this way, currently, documentary sources may include testimonies, oral sources, iconographic or audio-visual memories [18,24,25,26]; however, given the chronological setting in which we have developed this enquiry, we have opted for its recapitulation by the so-called “historical” method, set out and defended by Sánchez Granjel [27], by Salmon [28], by Romano [29] and by Laín Entralgo [30], which consists, as Arquiola indicated [31], in following the three phases of historical research proposed by Droysen in Germany, in the second half of the 19th century [32]: that is, the “heuristic”, or search for historical sources, their classification and evaluation through the auxiliary sciences of history; their “analysis and critical study”, both external or from authenticity and internal or from credibility; and finally, the “hermeneutic”, or interpretation phase of these historical sources [33].

As is well known, the purpose of science is to make known; the corresponding historical sources and disciplines that help the historian to evaluate them are known as heuristics, and in this case what concerns us is what Sánchez Granjel [27] called “written remains”, that is, those handwritten and printed sources in line with our objectives and purposes of study.

For this purpose, an exhaustive search and classification of the bibliographic collections of the archives and library of the Faculty of Medicine of Cadiz (currently transferred to the Historical Library of the University of Cadiz), the General Archive of the Indies, the General Archive of the “Álvaro de Bazán” Navy, the General Archive of Puerto Rico and the General Archive of the nation of Mexico were carried out. Subsequently, these sources were submitted to a strict analysis and critical study that determined their inclusion or exclusion in this research work, comparing them with different secondary sources and the critical bibliography [34] (p. 324). In this way, the location of the secondary sources and the critical bibliography was carried out by means of a general search pattern that included the title, summary and key words, in English and Spanish, related to the history of the vaccine and which entailed a qualitative systematic review of different printed texts contemporaneous with the chronology of the study; in this way, valuable historical press data were obtained which were subjected to the filter of the critical bibliography obtained from different databases, both national and international, among which it is worth noting, respectively, on the one hand, the CSIC (the Spanish National Research Council) databases, the Dialnet bibliographic website, Google Scholar and the catalogue of State Public Libraries, and on the other hand Web of Science and Scopus.

Finally, the rules that should govern the account of the knowledge obtained in the research were applied in order to achieve the proposed historiographical aims. For this reason, it was considered, as Laín Entralgo [35] stated, that the objective knowledge of history [36] should not be reduced to the mere positivist and scientistic historiographical exploitation defended by Sudhoff [37]. The historian must, in addition, apply an analytical–critical model to his or her research. This is what Topolsky [38] called “non-source-based” knowledge or, if preferred, hermeneutics or an unbiased interpretation of historical sources. In short, historiological research should emphasise not only a restrictive and poor view of history as an account of what is obsolete, but also show how science can acquire valid and useful knowledge over time, which, however, being products of human thought, are subject to possible changes in the light of new findings and subsequent judgements, in short, applying a model of approach to the study of the fundamental history of science based on the anthropological principles advocated by Sigerist, which is none other than the reflective and philosophical aspect of current knowledge based on its own past [39].

**Figure 1:**
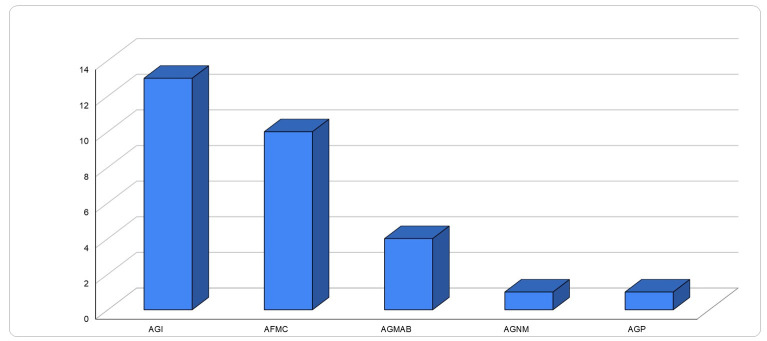
Number of manuscripts consulted in each of the archives. AGI: General Archive of the Indies (f = 13); AFMC: archives and library of the Faculty of Medicine of Cadiz (f = 10); AGMAB: General Archive of the “Álvaro de Bazán” Navy (f = 4); AGP: General Archive of Puerto Rico (f = 1); AGNM: General Archive of the nation of Mexico (f = 1).

A total of 29 handwritten documentary sources and 13 printed documentary sources were selected. These data made this study possible from a chronological, geographical and ethnographic point of view. It should be noted that 65.5% of the primary sources consulted were from the 19th century, contemporaneous with the historical facts, and the rest were documents from the 18th century corresponding to the records of the surgeons studied. A total of 44.8% of manuscripts consulted belong to the General Archive of the Indies, 34.5% to the archives and library of the Faculty of Medicine of Cadiz and 13.8% to the General Archive of the “Álvaro de Bazán” Navy; two documents were taken from the General Archive of Puerto Rico and the General Archive of the nation of Mexico (Figure 1).

## 4. Results and Discussion: The Correlation between People and Historical Facts

In line with our objectives and approaches, we present the results from the people identified as decisive to provide another historiographic look. Thus, all the surgeons identified in the story participated in some way in encounters and disagreements, and therefore in parallels between the universal and the local. In any case, the presentation of this correlation between people and historical fact allows us to assert that the vaccinal fluid was not only known in Spanish America and the Philippines before the landing of the previous surgeon and his expeditionaries, but also that it was used in many of the most important cities of those Spanish overseas departments before the arrival of the surgeon and his expeditionaries there, where generally it had arrived protected by glass, which, as we will show, was the frequent result of suspicions and confrontations between the expedition members and the doctors and the local authorities at the time of the mooring of the Maritime Expedition in these lands of overseas Spain.

### 4.1. Francisco Oller y Ferrer in Puerto Rico

Following the departure on 30 November 1803 of the corvette María Pita from the port of La Coruña and after its stay in Santa Cruz de Tenerife—between 9 December 1803 and 6 January 1804—the vaccine expeditionaries, led by Balmis, arrived on 9 February in the port of San Juan de Puerto Rico, in order to introduce the smallpox vaccine in Spanish America for the first time, as ordered by King Carlos IV.

**Figure 2:**
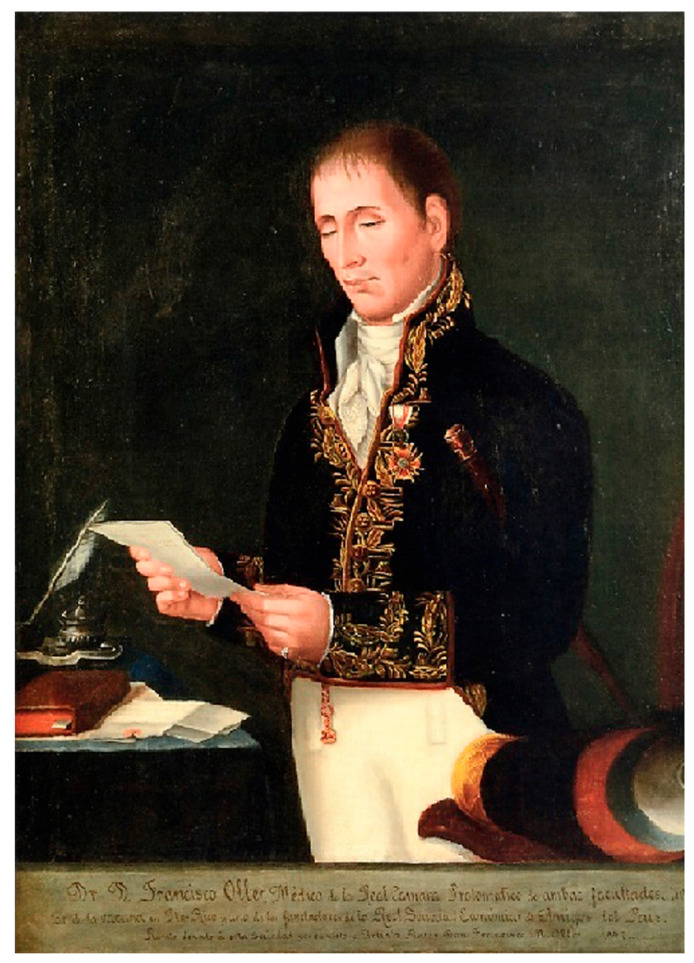
Portrait of Dr. Francisco Oller y Ferrer, 1847. Oil on canvas by Francisco Oller y Cestero (1833–1917, Puerto Rico), 43″ × 34″/109.2 × 86.3 cm. Col. Ateneo Puertorriqueño.

The expedition of Balmis was not well received by the Governor of Puerto Rico—Brigadier Ramón de Castro—nor by the senior surgeon of the Military Hospital, Francisco Oller y Ferrer [40,41] (Figure 2), who explained that a previous vaccination had already been performed. It should be noted that Balmis adopted a reactive stance, stating that in his opinion “Oller is inept, and his vaccinations have been ineffective” [42]. However, to the astonishment and incredulity of the expedition members, Francisco Oller y Ferrer showed them a large number of immunisations—more than 1500—that he himself had performed on patients he had previously vaccinated against the disease [10]; that is to say, for the Puerto Ricans, this vaccine was nothing new. Specifically, in 1803, Oller, aware of the existence of the vaccine, had urgently requested it from Governor Ramón de Castro [43] and Dr. Alexandre Mondeher, a resident of the Danish island of St. Thomas, who, in turn, had obtained it from the United States of America, which sent it to him on two occasions; the first shipment was unsuccessful, but the second, which Oller received on 28 November of the same year, was recorded in the General Archive of the Indies [44]. In this way, before the arrival of the Balmis Expedition, the island of Puerto Rico had become a pioneering centre for the diffusion of the vaccine in Spanish America, as it was rapidly dispatched to Cuba and Cumaná, today a province of the Bolivarian Republic of Venezuela, and from there to many other places in Spanish America [45] (p. 13).

Consequently, we can affirm, in principle, that the primary mover in the spread of the smallpox vaccine in the territories of Spanish America was not Balmis but, on the contrary, Francisco Oller y Ferrer [46], recognised in the Historical-medical Annals of Puerto Rico precisely for this fact, and whose hard and pioneering work in favour of the introduction and spread of the smallpox vaccine in American Spain has been thanklessly silenced in the Historical Annals of Medicine [47].

This reason explains why, only four weeks after its arrival in the Puerto Rican capital, obviously without having been able to achieve its objectives, that is, without having been able to spread the vaccine or train local surgeons in the technique of vaccination against smallpox because they already knew it, the Balmis Expedition decided to leave for the port of La Guayra in Venezuela on 12 March 1804. Although various setbacks in the voyage slowed down the journey to that port, endangering the potential of the vaccine, since in Puerto Rico there were hardly any unvaccinated children left [48] (p. 51), they finally decided to dock, with their participants, on 20 March 1804 in Puerto Cabello, already in the captaincy general of Venezuela [1] (p. 344).

### 4.2. Alonso Ruiz Moreno in Venezuela

Once arrived at the new destination, Balmis decided to divide the Expedition into three parties before reaching Caracas: the first was led by Balmis himself, which by land was to pass through Maracay to vaccinate the populations it encountered on its way; the second was commanded by Manuel García Grajales, which on board the ship Rambli was to reach La Guayra and proceed in the same way as the previous one; and finally, the third, under the command of José Salvany, was to remain in Puerto Cabello, on board the corvette María Pita, vaccinating the local population.

In this way, after completing the tasks entrusted to them, the three parties met on 30 March 1804 in the capital of Caracas to begin the vaccination campaign, which, in general terms, can be described as satisfactory and fruitful. On 23 April 1804, the Central Vaccination Board of Caracas was set up, which provided a stable structure for this procedure in this Captaincy General and, as the first, served as a model for the rest of the territories visited by the expedition during its journey.

However, once again, in admitting the above premise as absolute truth, history forgets the pioneering role played by Alonso Ruíz Moreno, a Navy surgeon, in introducing the smallpox vaccine in these Venezuelan lands [49], since this surgeon, prior to the arrival of the Balmis Expedition, had vaccinated the populations of Cumaná, Margarita Island and the governorship of Guayana with samples from Puerto Rico that had been sent to him by Francisco Oller [49] (pp. 233–237).

In any case, on the 9 April 1804, Francisco Xavier de Balmis, before leaving the Captaincy General of Venezuela, decided, in order to diversify efforts and give greater speed to the propagation of Jenner’s remedy in American Spain, to divide the Expedition again into two parties: the first was under his command, which on board the corvette María Pita would set sail for North America, and the second was under the command of José Salvany, which would set sail for South America on board the ship San Luis. For this reason, we must study the incidents of both departures separately.

### 4.3. Tomás Romay y Chacón and Bernardo Cózar y Delgado in Cuba

So it was that after an eventful voyage through the Caribbean, the corvette María Pita docked in Havana, Cuba, on 26 May 1804 to face, once again, the stark reality that the smallpox vaccine had also been successfully introduced on this Caribbean island [50,51], to be precise on 12 February of that year, by Tomás Romay y Chacón [52] and the Navy surgeon Bernardo Cózar y Delgado [53,54] (see [54], pp. 150–156; 185–191; and 352–360), following the landing of Carlos María Bustamante at the San Cristóbal station, who had left Aguadilla de Puerto Rico on the second day of that month after having vaccinated his ten year old son and two of his mixed-race maids the day before [55] (pp. 300–305).

In this way, Balmis, undoubtedly disillusioned by the results obtained so far in his Expedition, gave up his efforts to vaccinate in Cuba, leaving the port of Havana on 18 June of that year, not without facing various logistical problems due to the lack of children in which to preserve the vaccine, on his way to New Spain, only three weeks after his arrival in Cuba [4].

### 4.4. Miguel José Monzón y Farnel and José María Ledesma y Bórquez in Guatemala

After the inconveniences inherent in voyages through the Caribbean, Francisco Xavier de Balmis, together with the rest of the expeditionaries under his command, disembarked on 25 June 1804 at the port of Sisal, on the Yucatan peninsula, new American lands where again, to his displeasure, he was able to see how the smallpox vaccine had already been introduced by Miguel José Monzón y Farnel [56], surgeon of the Navy, who had begun his work, with the consent of Governor Benito Pérez, house to house, vaccinating more than one thousand, two hundred and twenty-seven people with vaccinal fluid from Veracruz [12], which is why, on the 29th day of that same month and year, just four days after the María Pita had landed on Yucatan soil, Balmis decided to move the party under his command to the city of Mérida, where, with the support of the local authorities, he imposed revaccination on its inhabitants, who had already been immunised by Monzón [56], an arbitrary measure decided by the director of the Expedition that led to a conflict with the local population and almost led to the loss of the vaccine due to the lack of popular support for the campaign [12].

Be that as it may, Balmis, after establishing the relevant Central Board of the vaccine in Mérida, decided that his nephew, Francisco Pastor de Balmis, would leave for the Captaincy General of Guatemala and, once the vaccine had spread there, would join him again, via Antequera de Oaxaca, in Mexico City.

In this way, Pastor left the port of Campeche, where the vaccine had also been introduced by Monzón [56], on 12 July 1804, to reach the city of Nueva Guatemala de la Asunción on 3 December of that year, where, to his surprise, the vaccine had also been introduced thanks to the efforts of different local doctors, among them Narciso Esparragosa, based on samples of active fluid from Trujillo that had been sent to that city on 16 June 1804 [57] (pp. 244–247) via ordinary mail, by José María Ledesma y Bórquez, surgeon of the Navy, so that by 23 June of that year there were already more than three or four thousand people vaccinated in this Captaincy General [1] (p. 317).

Far from being discouraged, Francisco Pastor, unlike his uncle, adopted a collaborative attitude with the local political and medical authorities, which doubtless favoured the establishment of the corresponding Central Vaccine Board which, in addition to preserving and propagating Jenner’s remedy in Guatemala, favoured its spread in San Salvador, León in Nicaragua and San Juan in Costa Rica [4], although its spread through these other lands did not take place until much later [58].

Balmis, on the other hand, having completed his mission in the port of Sisal, left on 19 July 1804 aboard the corvette María Pita for Veracruz, which he reached five days later. However, upon arrival, the director of the Expedition and his companions were ignored by the local population and authorities, since the vaccine had once again been introduced into this territory from samples of smallpox fluid from Havana, specifically from 29 March 1804 [10] (p. 61) by Bernardo Cózar and from 3 April of this year by Juan Ángel Pérez Carrillo, both Navy surgeons, which is why Balmis, seriously disappointed, decided to leave for Mexico City at the beginning of August of that year [59] (pp. 49–64).

### 4.5. Alejandro García de Arboleya and Antonio Serrano y Rubio in the Viceroyalty of New Spain

So, on 9 August 1804, he reached the town of Guadalupe, and although the director of the Expedition expected an official reception, organised by the viceregal authority, in accordance with the importance of his mission, this never took place, which, together with the lack of attention and the slowness with which he was supplied with the necessary material to carry out his vaccination work, led to the well-known and serious epistolary and verbal confrontation that Balmis had with the Viceroy of New Spain, José Joaquín de Iturrigaray. In this respect, it is worth noting the indifference shown at all times by Iturrigaray towards the members of the Expedition, alleging that their presence in the capital and in the territories under his command was not necessary [60], since the vaccine had been introduced, from 25 April 1804, from samples from Veracruz, by Alejandro García de Arboleya [61,62] and Antonio Serrano y Rubio [63], both surgeons of the Navy [64] (pp. 199–205).

Moreover, the attempts by Balmis to vaccinate the inhabitants of Mexico City and territories under his jurisdiction were in general useless, and he even had problems preserving the vaccine that he had to administer to ten soldiers in the garrison so that it would not be lost [65,66] (see [66], p. 207).

In this way, we can imagine that the director of the Expedition was overcome by events, and decided on 30 December 1804 to set sail, together with the rest of his expeditionary team and twenty-four Mexican children, for the Acapulco naval station and leave in the direction of the Philippines [67]. However, in something of a gesture of confrontation, Iturrigaray made his departure from the Viceroyalty of New Spain conditional on finding out whether or not the vaccine had been introduced in those islands; that is to say, he was not able to arrive at the Acapulco naval station until 27 January 1805 and to sail for the port of Manila aboard the ship San Fernando, alias the Magallanes, until 7 February of the same year, reaching the port of Manila after a rowdy journey full of reproaches from Balmis to the sailors on 16 April of that year [68].

### 4.6. Cristóbal Regidor in the Philippines

In the Philippines archipelago, Francisco Xavier de Balmis fell ill with dysentery, which led him to delay the start of the vaccinations until August 1805, being assisted in this task by Cristóbal Regidor, the first surgeon of the Navy and the doctor who was in charge of his condition until his departure [10]; given that his condition did not improve, the director of the Royal Expedition finally decided to leave, on 3 September of that year, for Spain—that is, only one month after his arrival in those lands—choosing for this the Portuguese route in order to avoid new confrontations with José de Iturrigaray, arriving at the port of Macao on 16 September of that year, where he also introduced the vaccine in the Chinese territory of Canton, and leaving there in February 1806 for Lisbon, on board the ship *Buen Jesús di Alem* [68].

In short, the vaccine was spread in the Philippines initially and mainly thanks to the work of the previously mentioned Cristóbal Regidor [68].

Balmis finally disembarked in the Portuguese capital on 14 August 1806 and was received, as already mentioned, on 7 September of the same year at the Court of Madrid by Carlos IV as a true national hero [68].

### 4.7. Ángel Hidalgo in Cartagena de Indias and Santa Marta in the Viceroyalty of New Granada

On the other hand, Salvany disembarked on 24 May 1804 in Cartagena de Indias, Colombia, where he was received with a tremendous welcome by the local political and financial authorities, and immediately began the first vaccinations, which were carried out on more than two thousand people of all ages and both sexes [68].

In addition, to complete his mission, on 10 July 1804, Salvany established in this area a corresponding Central Vaccine Board and sent samples of virulent lymph, contained in glass, to Panama, via Portobelo, and to Buenos Aires, via Riohacha, and then left, aboard a sampan, on the Magdalena River in the direction of Santafé de Bogotá [68].

Despite the above, we can assert that, paradoxically, the vaccine was already known in Cartagena de Indias before Salvany’s arrival; that is, we can affirm that the vaccine was not introduced and spread in a pioneering way in this territory by the deputy director of the Royal Expedition, but by Ángel Hidalgo [10], second surgeon of the Navy, specifically in Cartagena de Indias and the adjacent province of Santa Marta, as can be seen from the contents of his service record [10,68].

On the way to Santafé, the expeditionaries stopped in different coastal cities to carry out their vaccination mission, finally reaching the capital of New Granada on 17 December 1804, after Salvany had lost his left eye to ophthalmia [2] (pp.175–176).

On 8 March 1805, after having carried out fifty-six thousand, three hundred and twenty-four vaccinations, Salvany decided to leave Santafé. He divided the Expedition into two parties, one led by García Grajales which, after crossing the mountains of Quindío, was to aim for the cities of Neiva, San Sebastián de la Plata and Asunción de Popayán, and the other, led by Salvany himself, which would pass through the cities of Ybagué, San Jorge de Cartago, Trujillo, Llano Grande, Province of Chocó, Real de Minas de Quilichas and Asunción de Popayán, the last place being where he would join the other party [68].

Finally, on 27 May 1805, García Grajales and Salvany were reunited in the capital of Popayán, Colombia. By then, the deputy leader of the expedition, who had already suffered a previous episode of haemoptysis in Bogotá, had relapsed in his state of health, and recent news in the New Granadan town in Colombia of a serious smallpox epidemic in the Royal Court of Quito hastened his departure [68].

The Viceroyalty of New Granada was a vast region. In fact, it corresponded to contemporary Colombia, Venezuela, Panama, Ecuador and Guyana Esequiba.

Salvany once again divided the expeditionaries in his charge into two sections. One was under the direction of Manuel Julián García Grajales, which had to march towards the port of Santiago de Guayaquil; however, the plans to spread the vaccine along the Ecuadorian coast and send the vaccine to Panama were not possible due to the lack of funds in the Royal Treasury and the presence of the English Navy on Gorgona Island, in the bay of Santa Rosa de Atacames and at the cape of San Francisco, so this section had to set course for Quito by way of Malbucho or Carondelet without being able to fulfil its objectives, at least initially. On the other hand, the section under the charge of Salvany had to set off through the mountains towards the Quito region, passing through the towns of San Juan de Pasto, Tulcán, San Miguel de Ybarra and San Luis de Otavalo, finally meeting up again with Manuel Julián García Grajales in San Pedro de Cayambe, Ecuador [2]. However, despite the previously mentioned predictions, neither expedition met again, due to various misfortunes, until December 1806 in the Peruvian city of Lima [68].

### 4.8. Lorenzo Vergés in the Viceroyalty of New Granada

Be that as it may, what is certain is that once again, the official history takes the role played by another Spanish doctor in the introduction and propagation of the smallpox vaccine in the viceroyalty of New Granada (Colombia) prior to the arrival of the previously mentioned expeditionaries in Santafé de Bogotá; that is to say, Lorenzo Vergés established vaccination in New Granada using samples of bovine fluid contained in glass brought from the Iberian Peninsula before the arrival of Salvany [69]. The spread of the vaccine in this viceroyalty was unfortunately cut short by the early death of Vergés for the reason that he went to fight in America, but it is only fair to recognise that he alone deserves the honour of having been the true introducer of the vaccine in the capital of New Granada [4,70].

As we mentioned, José Salvany, still in a precarious state of health, in view of the news from Quito announcing an epidemic disaster caused by smallpox, decided to leave Popayán earlier and set course for the Royal Court, reaching the capital on 16 July 1805 and starting the vaccinations in the capital on 3 August of the same year, which he extended for two months, a time that also served him to recover from his illness, leaving, then, on 13 September of that year in the direction of Santa Ana de los Ríos de Cuenca, without García Grajales having yet disembarked in the Equadorian capital. Specifically, he arrived in the town four months later since he could not reach the port of Guayaquil until well into 1806, to the despair of the Guayaquil authorities who were about to start vaccinations using smallpox fluid from Portoviejo (Ecuador) [4].

### 4.9. Pedro Belomo y Ceballos in Lima and Santiago María del Granado in Upper Peru

The expeditionaries under the command of Salvany entered Lima on 23 May 1806, but to their surprise they found once again that the vaccine had been perfectly well established in the Peruvian capital since 23 October 1805 by Pedro Belomo y Ceballos, a Navy surgeon, on the basis of samples of smallpox fluid from Buenos Aires [45,71] (see [45], p. 15); given the irrefutable evidence, Salvany and his companions abandoned any vaccination project in the city and devoted themselves exclusively to establishing the corresponding Central Vaccination Board in that city, which was set up in mid-July of that year [45] (p. 15).

Once Salvany’s group was reunited with that of García Grajales, which reached the capital city of Lima in December 1806, the deputy director of the Expedition decided, on the one hand, that García Grajales should leave in March 1807 for Cuzco and the captaincy general of Chile, and on the other, that the rest of the expeditionaries, under his command, should head for Buenos Aires via Ica and the highlands of Arequipa, passing through Puno and La Paz, for which the latter began their journey on 28 January 1807. However, Salvany’s serious health problems put an end to his dreams in Cochabamba, in present-day Bolivia, where he died on 21 July 1810, that is, before he could reach the capital of the River Plate, and he received a Christian burial in the Church of San Francisco [72], though to his regret, once again, the vaccine had also been introduced in the Royal Court of Charcas and in the governorships of San Lorenzo de la Frontera, Moxos and Chiquitos [1,73] by Santiago María del Granado [74], surgeon of the Navy, who carried out a real vaccination campaign against smallpox in Upper Peru before the arrival of the expeditionaries [74] (pp. 935–965).

Therefore, none of the members of the philanthropic Royal Vaccine Expedition were able to reach the lands of the River Plate; however, fortunately for these Spanish Americans, Jenner’s remedy, as in many other of our overseas provinces, had already been introduced in this viceroyalty in a form unrelated to that of the landing of the Balmis Expedition in America [75].

### 4.10. Cristóbal Martín de Montúfar in the Viceroyalty of the River Plate

Specifically, on 5 July 1805, the three hundred and forty ton Portuguese slave frigate Rosa del Río entered Montevideo [76], transporting in its holds some vaccinal lymphs between glass [77] (pp. 14–15), which were delivered, among other things, to Cristóbal Martín de Montúfar [78,79] (see [79], p. 178), a surgeon from the Navy who successfully reproduced the vaccine pustule from these samples in four children living in Montevideo [58,80,81], and from here, in Buenos Aires, thanks to the expedition led by Juan Pérez García, also a Navy surgeon [79] (p. 178), to the rest of this viceroyalty [82] (pp. 8–9 and 19–26).

Finally, the vaccine was sent from an institutional position, through official channels between viceroys and using mail ships as transport, from the viceroyalty of the River Plate to the entire maritime route, once round Cape Horn, between the Captaincy General of Chile and Callao, and by land to other parts of the viceroyalty of Lima and the province of Cuzco; in this way, before the arrival of the Royal Philanthropic Vaccine Expedition to those territories, the city of Buenos Aires became the centre for the spread of the vaccine in South America [83] (p. 89).

On the other hand, when García Grajales, coming from Lima, arrived in Valparaíso at the end of December 1807, he found for the umpteenth time that the vaccine had already been applied in Santiago de Chile, since 9 October 1805 [83,84] (see [83], p. 77), specifically from samples of vaccinal fluid that the viceroy of the River Plate, the Marquis of Sobremonte, had sent between glass to the Chilean capital [85], the city where the vaccines had been initiated in the portico of the metropolitan Cabildo or Council by Fray Pedro Manuel Chaparro [85] (p. 78); even so, on 21 January 1808 García Grajales installed the first Central Board of the Vaccine of this Captaincy General in that Chilean city.

### 4.11. José María Gómez in Chile

The vaccine was brought to La Serena, capital of the Chilean region of Coquimbo, by José María Gómez, second surgeon of the Navy [10]. Just to add as a complement, his performance in the eradication of smallpox must have been very successful, as can be seen from the correspondence between Joaquín de Molina and Martín de Garay. Thus, Molina on 25 November 1809 wrote to him: “The result has been the most favourable, for those destitute peoples; because of the zeal of the physician Gomez, his application and disinterest in the visits of individuals, assistance and arrangement of the Hospital of San Juan de Dios, his care to acquire, preserve and spread the beneficial bovine fluid; it has been achieved with the greatest satisfaction of those inhabitants, the relief of their sick and above all stopping the dreadful progress of smallpox more fearsome in that country than in any other. For all of which, the referred Professor Don José Gómez, has become a real merit, worthy of the Supreme Government, to attend him with a distinction or employment, providing his career, to whose effect I make His Excellency this exhibition, begging him to elevate it to the Sovereign Board” [10].

## 5. Final Considerations

The vision described and analysed constitutes a decisive correlation for the construction of a more coherent and comprehensive history of this pandemic. In this way, we want to highlight the importance of delving into the analysis of historical documents and primary sources to better understand local actors and dynamics involved in public health. As we have been able to verify in two past and independent times of history, the former overseas dominions of Spain benefited from Jenner’s remedy; so, the History of Medicine has given an ample account of one of them, linked to the Royal Philanthropic Expedition of the vaccine, as an indelible and unparalleled memory at world level, but it is unjustly silent with respect to the other, leaving it lacking in fair and impartial recognition and the perpetual record in its pages.

Thus, historical facts show that before the arrival of Balmis the vaccine substance was introduced in these countries thanks to the initiative of several surgeons. Therefore, the work of these professionals must transcend and be duly recognised. They were: in Puerto Rico, Francisco Oller; in Cartagena and Santa Marta in Colombia, Ángel Hidalgo; in Venezuela, Alonso Ruiz; in Cuba, Tomás Romay and Bernardo de Cózar; in the Viceroyalty of New Granada (Colombia), Lorenzo Vergés; in Guatemala, Miguel José Monzón and José María Ledesma; in the Viceroyalty of New Spain, Alejandro García Arboleya and Antonio Serrano; in Peru, Pedro Belomo; in Río de la Plata, Cristóbal Martín de Montúfar; in the Chilean region of Coquimbo, José María Gómez; and in the Philippines, Cristóbal Regidor. Most of them trained at the Medical–Surgical School of Cadiz [40,41,57,74,77,78,79,80,81,82,83,84,85,86,87,88,89,90,91,92,93].

Despite what has been said, the work carried out by the expeditionaries under the command of Balmis in the fight against smallpox overseas should not be underestimated; in fact, the Royal Philanthropic Expedition of the vaccine is considered the first international health expedition in the History of Medicine, but, equally, if the text written by Francisco Xavier de Balmis has had all the impact it deserves, it is also relevant to add to it those other initiatives, not only because they were prior to his own, but because they were the fruit of the fervour and zeal of those humble surgeons of the Spanish Navy.

## Data Availability

All data are included as part of the manuscript.
